# **Changing the Discourse in Ambitions Towards Universal Health Coverage: Lessons From Australian Primary Healthcare** Comment on "Universal Health Coverage for Non-communicable Diseases and Health Equity: Lessons From Australian Primary Healthcare"

**DOI:** 10.34172/ijhpm.2021.165

**Published:** 2021-12-01

**Authors:** Sarah J. Simpson, Victoria Saint, Kayvan Bozorgmehr

**Affiliations:** ^1^Independent Consultant, Lyon, France.; ^2^The University of New South Wales, Sydney, NSW, Australia.; ^3^Department of Population Medicine and Health Services Research, School of Public Health, Bielefeld University, Bielefeld, Germany.; ^4^Section for Health Equity Studies & Migration, Heidelberg University Hospital, Heidelberg, Germany.

**Keywords:** Australia, Equity, Non-communicable Diseases, Financial Risk Protection, Universal Health Coverage, Structural Determinants

## Abstract

While Australia’s health system has reached universal health coverage (UHC), recent scholarship points to its strengths and identifies ways it could be more effective and equitable, especially for tackling non-communicable diseases (NCDs). Building on the Australian experience, we add to these perspectives and present pertinent lessons for the quest towards UHC, and for policy-makers globally with regard to NCDs. Potential lessons include: the need for (*i*) vigilance – UHC requires ongoing monitoring and evaluation of not only financial risk protection but non-financial barriers and impacts such as forgone care; (*ii*) investment and action now on structural determinants of NCDs and related inequalities to avoid potentially higher (fiscal, social and health) costs in the longer term; and (*iii*) the opportunity for policy-makers globally and nationally to revisit their ambitions for UHC to include population health policies/ programs beyond essential health services that are required for healthier, more equitable and thriving societies.

 In their recent policy analysis, Fisher et al^1^ outline ways in which Australia’s universal healthcare system can be strengthened for more equitable management and primary treatment of non-communicable diseases (NCDs). As well as presenting evident challenges, they point to more fundamental issues such as the system’s deep roots in a pathogenic and predominantly biomedical paradigm.^2^ These roots run deep in many national healthcare systems, including Australia, and in many agencies driving global health strategy and normative guidance. Consequently, policy discourse on improving universality and equitably tackling NCDs often ends up focusing on discussions around barriers to healthcare and treatment services, and the even narrower focus on financial risk protection by means of insurance mechanisms or companies. This curtails opportunities for broader dialogue and policy-making processes that translate into coherent, sustained policy action anchored to primary healthcare (PHC) principles to create and promote health equitably and in a more comprehensive way – consistent with the original (1978) and renewed (2018) Astana Declarations on PHC.^3^

 In this commentary we add to the perspectives of Fischer et al^1^ and draw out pertinent lessons from the Australian experience for the quest towards universal health coverage (UHC) and for policy-makers with regard to NCDs. Given the impact of NCDs for all countries, with 80% of NCDs occurring in low- and middle-income countries,^4^ we consider lessons that may be relevant to all contexts from those in the early stages of designing a system for UHC through to policy-makers in countries with well-established systems. The starting point for our reflections is the *status quo* in monitoring for UHC, and the need to move beyond financial risk protection to inform policy about factors impeding access to healthcare. We then outline the need to invest in action on the structural determinants of health, and close with reflections on the costs of inaction to derive lessons for policy-makers.

## Monitoring for UHC Beyond Financial Risk Protection to Non-financial Factors

 Discussions on ensuring universality and equity in UHC systems, have increasingly tended to focus on financial risk protection measured in terms of out-of-pocket (OOP) and catastrophic expenditure. Fisher et al^1^ point to the different policy strategies put in place in Australia to improve PHC including strengthening equity in terms of financial risk protection. Theoretically, no OOP costs or fees should be incurred for a primary care visit if the general practitioner charges the national Medicare Benefits Scheme (MBS) fee.^5^ In practice, while 86% of general practitioners in Australia in 2016-2017 billed using the MBS fee, OOP expenditure was 16.5% of total health expenditures with primary care OOP payments forming 68%.^5^ The Original and Extended Medicare Safety Nets are in place to cover OOP and financial expenditure over specific cost thresholds and for eligible out-of-hospital services (see Box 1).^5^ However, individuals and households on limited income, even if not in the most disadvantaged quintile or decile, may not be able to afford the initial expenditure required before they reach the limit to be eligible for the safety net assistance. These population groups may potentially forego care to avoid incurring debt, putting their health and well-being at risk, and potentially requiring more advanced treatment later on with corresponding higher costs for the health system.

**Box 1. Original and Extended Medicare Safety Nets**

The Original Medicare Safety Netcovers out-of-hospital costs above AU$ 481 (US$ 370) at 100% of the MBS fee.^5^ Since 2004 the Extended Medicare Safety Netprovides an additional rebate for Australian families and singles who incur OOP costs for Medicare eligible out-of-hospital services.^6^When the annual threshold of OOP costs is reached, Medicare pays for 80% of any future OOP costs for out-of-hospital Medicare services for the remainder of the calendar year. There are two thresholds for the Extended Medicare Safety Net(indexed by the Consumer Price Index on 1 January each year) and as follows: (1) AU$ 697 (US$ 537)for Commonwealth concession cardholders, including those with a Pensioner Concession Card, a Healthcare Card or a Commonwealth Seniors Card, and people who receive Family Tax Benefit (Part A); and (2) AU$ 2184 (US$ 1682)for all other singles and families.^6^

Abbreviations: MBS, Medicare Benefits Scheme; OOP, out-of-pocket.

Forgone care, a measure of non-realized access to healthcare as opposed to healthcare utilization,^7^ is rarely monitored on a regular basis globally when measuring UHC system performance. The Organisation for Economic Co-operation and Development biannual *Health at a Glance* reports information on unmet needs with the 2011, 2015 and 2017 reports indicating little change in the percentage of Australians with unmet care needs on either above average income 12%-14% or below average income 21%-24% during these three reporting periods.^8-10^

 Underlining the fact that there continue to be considerable challenges in terms of unmet need, a recent study showed “*[…] that Australia’s universal health system appears not to safeguard the poorest people in society […] against the financial hardship with accessing healthcare*”^11^(p. 6). It highlights that households with lower income are at higher risk of losing their employment or livelihoods due to (un- or under-treated) chronic disease or illness linked to their inability to meet OOP.^11^ In Australia there is no agreed level on what proportion of household income makes expenditure on healthcare “catastrophic”^11^ and physiotherapy, dental and allied health services are not covered by Medicare,^1^ raising questions on its universality, ie, the depth of coverage, in the system. Ongoing monitoring of the distribution and impoverishing consequences of OOP is hence needed, rather than assuming that financial risk protection is an inevitable or natural outcome having a universal health system.^11^

## The Need for Investment and Action on Structural Determinants of Health

 There is a strong connection between improved education and economic development and health, and the reverse with low socioeconomic status leading to chronic illness and reducing household incomes.^4,12-14^ There has also long been evidence that health inequalities have direct economic and social costs to individuals and wider society.^12,14^ Research for the European Union in 2010 estimated the health consequences related to socio-economic inequality included more than 700 000 deaths per year and 33 million cases of ill health in the European Union with related healthcare and social security costs.^14^ Inequality related losses to health were found to reduce labor productivity and health inequality related welfare losses were estimated at €980 billion per year or 9.4% of gross domestic product.^14^In 2012, Catholic Health Australia commissioned research to look at what might be achieved through acting on the recommendations of the World Health Organization (WHO) global Commission on Social Determinants of Health in its 2008 report and the costs of inaction.^12,13^ The research highlighted significant cost savings to health and social care systems and positive gains for individuals, households, communities and the wider population in terms of improved health and well-being.^12^ For example, half a million Australians could avoid a chronic illness; 60 000 less people would need to be admitted to hospital annually (saving $2.3 billion in hospital expenditure), 5.3 million fewer Pharmaceutical Benefit Scheme scripts would be filled each year (with annual savings of $184.5 million) and 170 000 extra Australians could enter the workforce, creating $8 billion in extra earnings.^12^

 Australia’s UHC system including the PHC component has been in place since the mid-1980s. Like many established or older UHC systems, it was designed at a time when NCDs were not central concerns or goals for population health. At the same time, however, Australia was quickly becoming a global leader in tobacco control through policy and legislative action including being one of the first countries to prohibit the advertising of cigarettes on television and radio (1976).^15^ This was further enabled by widespread civil society action against tobacco companies.^15^ Consequently Australia has one of the highest tobacco taxes in the world as part of a very restrictive and comprehensive approach to tobacco control.^15^ In April 2010 there was a one-off 25% increase in the price of cigarettes followed by a 12.5% increase from December 2013 and then each September thereafter until September 2020. After September 2020, this meant that the average 20 pack of cigarettes costs around AU$ 35.^16^

 Evidence demonstrates the success of tobacco taxation in Australia and other settings in terms of reduced consumption, morbidity, mortality and healthcare costs.^17^ This however has not led to widespread commensurate action on other unhealthy products such as processed food, salt or sugar or alcohol.^18-19^ Despite increasing rates of chronic and complex conditions in Australia, health system action to tackle NCDs remains largely focused on disease and direct behavioral risk factors via primary (medical) care and treatment.^1^ Globally, there is a similar pattern, with a limited number of national and sub-national authorities implementing sugar or salt taxes, including Finland, France, Hungary, Latvia, Mexico and Portugal.^20^ Despite the existing evidence, including modelling studies of the impacts of taxes on health and health costs,^18-19^ the politics of implementing fiscal measures to control NCDs at population level are incredibly challenging. A case in point is Denmark, which introduced and later repealed its tax on saturated fat.^20^ Nevertheless, countries implementing such taxes are evaluating their efforts, strengthening the evidence-base and providing substantial opportunities for global exchange about the key lessons from their implementation.

 Informed by these developments, countries that are currently designing or redesigning their health systems for UHC and or their NCD strategies have an opportunity to also gear their systems towards tackling upstream and structural determinants of NCDs. Most NCD risk factors (sugary drinks, fat and salt in processed food, tobacco) share common structural and commercial determinants^[1]^. For example, transnational corporations penetrating global markets through unregulated trade policies.^22^ These upstream determinants often also act as or interact with upstream drivers of other population health issues, including infectious diseases, in ways that can create a “double-burden of disease” or dynamic syndemic for countries. For example, the risk of acquiring tuberculosis (TB), dying from TB during treatment, or relapsing after treatment is higher among people with diabetes.^23^ As such, the global increase in diabetes is a concern for TB control, particularly in countries with a high burden of TB.^24^ Furthermore, the complex interactions between NCDs, infectious diseases and their common structural drivers are playing out within and dynamically interfacing with environmental factors and climate change.^25^ Tackling structural determinants of NCDs, such as the production and unregulated sale of unhealthy commodities, might be done synergistically by seeking to address other related health, environmental and social challenges. For example, preventing and reducing air pollution presents a critical opportunity for synergistic action in relation to NCDs given the connections between ambient air pollution and cardiovascular and respiratory disease, and cancers.^26^

###  Widening the Policy Space of UHC Ambitions 

 What follows from the above for policy-makers in other countries is that there are costs of narrowing the focus for action on NCDs to that of clinical care and ignoring or underinvesting in action on wider determinants of NCDs and related inequalities. The expectation is not that healthcare or service providers are directly responsible for action on upstream determinants of NCDs but should advocate and support efforts to widen the policy space for action. Fisher et al however point to the challenges nowadays in getting a wider view of prevention on the policy and health systems table, describing the predominantly biomedical framing that underpins funded services and care to a focus on individual lifestyle change and “… marginalizing strategies that address the social determinants by creating healthy social conditions.”^1^ Key actors exercising policy influence in this context in Australia are medical professionals’ representative organizations, private health insurance companies, and food and alcohol industry corporations.^1^ This shrinks the available policy space for upstream action and primary prevention and diverts both our gaze and the resources (financial and human) away from looking at what we could do to reduce the number of people requiring health services and care for NCDs and reduce the increasing costs at healthcare level through improved prevention. This brings us back to the earlier question – what are the costs of *in*action on the structural and commercial determinants of NCDs. Investment in actions to address structural and commercial determinants, for example, to reduce unhealthy consumption, unfair trade arrangements or to improve air quality can promote, protect, and improve health for a greater proportion of the population. From a universality perspective such measures need to be implemented in ways that are progressive and ameliorate rather than exacerbate existing inequalities. This calls for a structural and systems approach to tackle the impending morbidity crisis from NCDs in a way that is equity oriented.^27^

 If we are to realize the wider ambitions of UHC and the SDGs then a different approach is needed.^28^ The SDGs are intentionally interconnected,^4,29^ with the original intent of UHC going beyond healthcare *per se*. The most recent Astana Declaration^3^ is the latest in a long line of global policy documents underlining the importance of action on social, economic, cultural, commercial and environmental determinants of health.^27,28^ This means we need to look at “[…] fixing the broken system rather than the people in the system,”^27^ or to what is needed to create healthier and more equitable societies.^2^ This does not preclude the need for strengthening equity and coverage through PHC for all people with chronic conditions. Indeed, this is critical and two of WHO’s triple billion targets^29^ – those on UHC and healthier populations – speak to the need for action on both fronts.

 The delicate balancing act for policy-makers is to design and advance UHC systems in ways that simultaneously (*a*) act on the social and structural determinants to prevent NCDs, (*b*) ensure pooling and protection of funds for primary prevention and promotion activities in health and related social sectors, and (*c*) ensure the basket of ‘essential’ health services includes preventive, promoting and primary care level actions on NCDs and risk factors. This evidence is not new^27^ yet we need to keep revisiting it for some reason. Australia’s experience presents an opportunity for policy-makers globally to consider not only how to strengthen healthcare for NCDs but also how to prevent people developing NCDs in the first place; and how some of the ways in which the design of a health system and its mechanisms can make this difficult.^1^ In considering the costs of such action, research from Australia such as that by Fisher et al^1^ and others can provide a counterpoint in demonstrating the longer term costs – human, financial and systems – of inaction and underinvestment in tackling the structural determinants of NCDs and primary prevention.

 The coronavirus disease 2019 pandemic has generated massive disruptions to health, social, economic, and political systems worldwide and prompted widespread and deeply critical reflection about the State and rate of progress in tackling the complex web of social, environmental and governance challenges that humanity is facing. Many hope that these disruptions will generate the necessary political and public will to finally tackle the deeper structural transformations widely committed to but rarely acted upon. Within this space is a real opportunity for policy-makers globally and nationally to revisit their ambitions for UHC to include population health policies and programs beyond essential health services that are required for healthier, more equitable and thriving societies.

## Ethical issues

 Not applicable.

## Competing interests

 Consultancy fees for two authors (SJS and KB) but not used for producing the commentary, and commentary only draws on existing publications and not from these contracts; one author has a patent (KB); and two authors (SJS and VS) know two of the authors on the Fisher et al paper (S Friel and F Baum) but have no financial or current working relationship with them in the past 36 months.

## Authors’ contributions

 SJS conceptualised and designed the commentary initially, thereafter all three authors made equal contributions for the final product.

## Authors’ affiliations

^1 ^Independent Consultant, Lyon, France. ^2^The University of New South Wales, Sydney, NSW, Australia. ^3^Department of Population Medicine and Health Services Research, School of Public Health, Bielefeld University, Bielefeld, Germany. ^4^Section for Health Equity Studies & Migration, Heidelberg University Hospital, Heidelberg, Germany.

## Endnote


^[1]^ Structural determinants are the political, legal, and economic determinants with social norms and institutional processes that shape the distribution of power and resources determined by the conditions in which people are born, grow, live, work, play and age. Commercial determinants of health are the conditions, actions and omissions by corporate actors that affect health negatively or positively.^21^
